# Inter-hospital cardiorespiratory telemonitoring of newborns and infants: a wellworking example of a hub and spoke network

**DOI:** 10.1186/s13052-022-01407-2

**Published:** 2023-01-13

**Authors:** Cinzia Arzilli, Monica Annunziata, Carola-Maria Ernst, Marta Peruzzi, Chiara Macucci, Saverio Pochesci, Niccolò Nassi

**Affiliations:** 1 Meyer Children’s Hospital IRCCS, Sleep Disorder Breathing and SIDS Center, Viale Pieraccini, 24, Florence, 50139 Italy; 2 Department of Paediatrics, Meyer Children’s Hospital, University of Florence, Florence, Italy

**Keywords:** Telemedicine network, Hub-and-spoke system, Apnoea of prematurity, Apparent life-threatening events

## Abstract

**Background:**

Patients who experience cardiorespiratory events usually have to be moved to specialized centers to perform cardiorespiratory studies. To avoid the transfer of these patients to specialized centers, a network has been created based on an interchange system, where the recordings were uploaded in unspecialized centers (spokes) and downloaded by the Sleep Disorders Breathing (SDB) Center (hub) to be analyzed.

**Methods:**

The inter-hospital network was established in November 2008. Initially only 3 non-tertiary hospitals in the Tuscany Region joined the network. Currently, 12 Tuscany hospitals are included.

**Results:**

From November 2008 to December 2020, 625 recordings were collected belonging to 422 infants.

No recurrent life-threatening episode or infant death occurred in the study population and none of the infants needed to be readmitted or be moved to a tertiary center, except infants who underwent home monitoring.

The discharge diagnoses belong to the following categories: apnoea, respiratory problem of the newborn, syncope, gastroesophageal reflux, altered consciousness, transient loss of consciousness and cyanosis.

**Conclusions:**

This study shows that the inter-hospital network is an efficient system that allows accurate and safe management of infants at risk for apnoea, bradycardia, and hypoxemia to remain in unspecialized centers, avoiding unnecessary transfers of patients and over – hospitalizations.

## Background

The assessment of cardiorespiratory patterns in symptomatic preterm newborns and infants with a clinical history of higher risk events may represent an arduous challenge.

Complete polysomnography (PSG) is the gold standard in the diagnosis of Sleep Disorders Breathing (SDB).

Due to the complexity and poor availability of PSG [1, 2], multichannel overnight recordings may represent a simplified method for the evaluation of cardiorespiratory events. Moreover, direct bedside observation of cardiorespiratory events performed by the nursing staff may cause an under/overestimation of significant events leading to incorrect clinical management [3, 4].

Preterm newborns (Pt) are frequently prone to apnoeas, bradycardias, and/or oxygen desaturations; their duration and clinical features should be analyzed to evaluate if they are potentially life-threatening or harmful to the integrity of the central nervous system.

Documented cardiorespiratory monitoring is therefore useful in the management of symptomatic Pt that usually requires a time interval of 3 – 5 – 7 event-free days to be considered eligible for the discharge [5].

Multichannel overnight recordings may be useful in the clinical assessment of infants with a history of events previously identified as “ALTE” (Apparent Life-Threatening Event), an acronym coined in 1986 to define a frightening episode characterized by the variable combination of apnoea, changes in skin color, and muscle tone [6].

In 2016 the American Academy of Pediatrics (AAP) proposed to change this acronym with the new term “Brief Resolved Unexplained Events” (BRUE) indicating only low-risk events [7]. The Italian Guidelines [8] adopted the new acronym BRUE maintaining the term ALTE to refer only to idiopathic higher-risk cases that may need a cardiorespiratory recording for 24/48 h after the event because in this period there is a greater risk of recurrence [9–12].

In this study, cardiorespiratory recordings belonging to subsequent siblings (SS) of Sudden Infant Death Syndrome (SIDS) victims and newborns who experienced a neonatal ALTE (n-ALTE) or a Sudden Unexpected Postnatal Collapse (SUPC) were also included.

Despite the relevance in studying cardiorespiratory patterns in newborns and infants, the implementation of multichannel recordings in non-tertiary centers may be difficult, especially in data interpretation.

An inter-hospital network was developed to simplify this procedure: it was structured as a hub-and-spoke system finalized to share cardiorespiratory files, recorded and uploaded on a website by non-specialized centers (spoke), and downloaded to be analyzed in the tertiary specialized center (hub).

In conclusion, this study aims to evaluate network efficacy to prompt document cardiorespiratory events in newborns and infants hospitalized in non-tertiary centers. The main advantage of this system is represented by moving traces instead of patients improving the clinical management.

## Methods

### The network

The inter-hospital network was established in November 2008 including 3 non-tertiary hospitals in the Tuscany Region. Currently, 12 hospitals are included and reported in Table 1.

**Table 1:** Number and percentage of recordings from spoke centers

SPOKE CENTERS		
	**No**	**Percentage**
**Massa Carrara**	190	30,4
**Prato**	103	16,5
**Pistoia**	64	10,2
**S. Giovanni di Dio Hosp. (Florence)**	54	8,6
**Livorno**	44	7
**Careggi Hosp. (Florence)**	43	6,9
**Empoli**	39	6,2
**S. Maria Annunziata Hosp. (Florence)**	37	5,9
**Arezzo**	28	4,6
**Siena**	13	2,1
**Pisa**	10	1,6
	**625**	

The project aimed to obtain cardiorespiratory recordings from non-tertiary centers avoiding the patients’ transfer and the discomfort for families.

Therefore, a dedicated website that allows the upload and sharing of recordings was created. The SDB Center staff trained the members of spoke centers on this procedure and monitor utilization. This training session was repeated each time a new hospital joined the network.

A secured professional area on the website was reserved only to professionals through a specific username and password to guarantee patients' data privacy [13, 14].

A formed sheet reporting cardiorespiratory alarms (bradycardia, apnoea, desaturation), the circumstances of the alarms (i.e. crying, feeding, sleep), and the intervention eventually performed (i.e. tactile stimulation, resuscitation, O2 administration) was given to the nursing staff to compare their documentation with tracings recorded by the monitor.

The communication between hub and spokes of new cases was performed by telephone and/or e-mail.

From January 2022, the system will be converted to teleconsultation to guarantee greater patients privacy protection and shorter response times.

### Patients’ characteristics

Four hundred and twenty-two patients (235 males; age 50.2 days ± 75.3) at risk for adverse cardiorespiratory events were evaluated. Two hundred (47.4%) were infants with a clinical history of ALTE, 130 (30.8%) Pt with apnoea of prematurity, 78 (18.5%) newborns with a clinical history of n-ALTE and 14 (3.3%) SS. Patients’ characteristics are summarized in Table 2.

**Table 2:** Number and percentage of patients with a clinical history of Apparent Life-Threatening Events (ALTE), Prematurity (Preterms), Neonatal ALTE (n-ALTE), and Subsequent Siblings of SIDS victims (SS); Gender; Mean ± SD Age expressed in days

PATIENTS CHARACTERISTICS				
	**No. of patients**	**Percentage**	**No. of Male/Female**	**Mean Age (days) ± SD**
**Total**	422	100	235/187	50.2 ± 75.3
**ALTE**	200	47.4	100/96	69.6 ± 88.1
**Preterms**	130	30.8	83/47	24.9 ± 38.2
**n-ALTE**	78	18.5	42/36	9.3 ± 17.2
**SS**	14	3.3	10/4	6.2 ± 12.4

### Devices and monitoring techniques

Patients were monitored with Getemed VitaGuard 3100® monitors. This cardiorespiratory monitor records and displays multiple traces (thoracic impedance waveforms, respiratory rate, heart rate, ECG-QRS waveform, oxygen-saturation pulse waveform, and pulse rate) and allows adjusting thresholds for acoustic alarms and silent events.

The devices were set according to patients’ age. All alarms and silent event traces were visually analyzed and classified as central apnoea, central apnoea with bradycardia, central apnoea with desaturation, isolated bradycardia, tachycardia, isolated desaturation, and periodic respiration with and without hypoxemia [15, 16]. The device has also a specific algorithm that allows identification of periodic breathing. This feature is crucial in the diagnosis of apnoea of prematurity.

The telemonitoring system works by connecting the cardiorespiratory monitor to a PC via a USB port. The recordings are downloaded and stored in the dedicated software and then uploaded in the operators’ area of the website. The tracings were scored by two senior clinical investigators with proven expertise in the assessment of cardiorespiratory traces, belonging to the SDB Center (hub).

The system is asynchronous: the downloading and scoring of the traces and the generation of the reports were sent back within the next 8 hours from request.

## Results

From November 2008 to December 2020, 625 recordings were collected belonging to 422 infants from the spoke centers, as reported in Table 1. There was a steady increase of requests from 2 cardiorespiratory files (2008) to 118 files (2020). The duration of recordings performed in the hospitals was variable, from a minimum of 24h to a maximum of 96h.

In particular, 48 recordings lasted 24 hours, 363 recordings 48 hours, 160 recordings 72 hours and 10 recordings lasted 96 hours. Long monitoring duration was ranged from 7 days to 1 month from discharge.

There wasn’t any relevant alarm trace tracing in 357 (57.1%) out of 625 recordings. In the remaining recordings, both isolated and combined events have been detected as reported in Table 3.

**Table 3:** Events characteristics

EVENTS CHARACTERISTICS					
(1) **ISOLATED EVENTS**					
	**ALTE**	**n-ALTE**	**Preterms**	**SS**	**TOT**
PB	6	2	3	0	11
CA	1	1	6	0	8
B	7	6	4	0	17
H	10	3	28	2	43
T	1	0	1	0	2
(2) **COMBINED EVENTS**					
	**ALTE**	**n-ALTE**	**Preterms**	**SS**	**TOT**
A + B	0	0	5	1	6
A + B + H	2	2	11	1	16
A + H	1	0	3	0	4
A + T	0	1	2	0	3
B + H	1	2	3	0	6
PB + H	5	1	10	0	16

(2)—Number of isolated cardiorespiratory events in each group: Periodic Breathing (PB), Central Apnoeas (A), Bradycardias (B), Hypoxemias (H), Tachycardias (T). (3) – Number of combined cardiorespiratory events in each group: Apnoeas and Bradycardias (A + B), Apnoeas Bradycardias and Hypoxemias (A + B + H), Apnoeas and Hypoxemias (A + H), Apnoeas and Tachycardias (A + T), Bradycardias and Hypoxemias (B + H), Periodic Breathing and Hypoxemias (PB + H)

The Ninety-nine infants that underwent home monitoring belonged to the following categories according to the recommendation of Italian Guidelines [8] and literature [17]: 1) infants who have experienced an ALTE; 2) infants with tracheostomies or anatomic abnormalities; 3) infants with neurologic or metabolic disorders; 4) infants with chronic lung disease.

No recurrent life-threatening episode or infant death occurred in the study population and none of the infants needed to be readmitted or be moved to a tertiary center, except infants who underwent home monitoring. Thirty-seven underwent xanthine therapy.

The discharge diagnoses belong to the following categories: apnoea, respiratory problem of the newborn, syncope, gastroesophageal reflux, altered consciousness, transient loss of consciousness and cyanosis.

## Discussion

Telemedicine potentially allows achieving equity and improving the quality of health care through easier accessibility and a cost reduction in any field of medicine [18–23]. The analysis of cost reduction is not a goal of this study, so more detailed data on telemedicine network cost analysis should be performed.

Previously the efficiency of a telemonitoring system carried out directly from the patients’ home in home-monitored infants [24] was demonstrated; in the present descriptive study the usefulness of the tele-transmission of cardiorespiratory recordings of infants and newborns at risk for apnoea, bradycardia, and/or desaturations, within hospitals belonging to a hub-and-spoke network was retrospectively evaluated.

Our data shows that all ALTE infants admitted to non-tertiary centers may undergo timely documented monitoring and be discharged directly from their hospitals after performing the first-line examination algorithm, according to national guidelines for ALTE [8].

The cardiorespiratory overnight recordings are also useful for clinical management of symptomatic preterms, especially in planning the correct therapeutic approach and discharge time, in order to reduce the number of transfers in tertiary centers and rehospitalizations [8, 15].

The data interpretation performed by skilled scorers allows to individuate the real cardiorespiratory patterns and discover underlying conditions avoiding re-admission due to an unsafe discharge.

## Conclusions

This telemonitoring network resulted in a functional system that partially close the gap between specialized and non-specialized hospitals in performing cardiorespiratory recordings, ensuring prompt and thorough management of newborns and infants at risk of potentially harmful cardiorespiratory events.

In particular, the strong impact of our telemedicine network in supporting non-tertiary centers is confirmed by the lack of re-admissions due to a safe event-free period before the discharge and by the absence of patients transferred in tertiary hospital to perform sleep studies.

Moreover, due to the increasingly number of the hospitals that joined the network over the year, the next step will be to promote the inclusion of other hospitals to guarantee similar management of cardiorespiratory events in non-tertiary centers.

## Data Availability

The data that support the findings are available from the corresponding author on reasonable request.

## Abbreviations

PSG: Polisomnography
SDB: Sleep Disorder Breathing
Pt: Preterm newborns
ALTE: Apparent Life-Threatening Event
AAP: American Academy of Pediatrics
BRUE: Brief Resolved Unexpected Events
SS: Subsequent Siblings
SIDS: Sudden Infant Death Syndrome
n-ALTE: Neonatal Apparent Life-Threatening Event
SUPC: Sudden Unexpected Postnatal Collapse
ECG-QRS: Electrocardiogram-QRS
